# Observations in the Department of Pathology and Pathological Anatomy

**Published:** 1838-01

**Authors:** 


### ART. XIV.

*Beobachtungen auf dem Gebiete der Pathologie und Pathologischen Anatomie.* Gesammelt von Dr. JOH. FRIED. HERM. ALBERS, Professor der Medizin an der Rheinischen Friedrich-Wilhelms Universität, in Bonn, &c. Erster Theil.—*Bonn*, 1836. 8vo. pp. 204.

*Observations in the Department of Pathology and Pathological Anatomy.* Collected by Dr. J. F. H. ALBERS, Professor of Medicine in the Rhenish University of Frederick-William, at Bonn. Vol. I.—*Bonn*, 1836. 8vo. pp. 204.

IT is the ardent wish of Dr. Albers, as it is that of most authors, that his work may be esteemed a valuable contribution to the science of which he treats. His observations certainly afford proofs of great reading and research, and are in themselves both interesting and instructive; but they are too much loaded with quotation, and too deficient in originality, to warrant their being brought forward as a new work in a department in which medicine is so rapidly advancing.

The crying sin which pervades the whole book is a desire of novelty without its reality, which has led our author to invent hypotheses, in place of drawing inferences from facts. In many instances his speculations are so purely fanciful, that they seem to have been contrived more for the purpose of differing from other authors, than from any intention of conveying instruction.

Dr. Albers's first observation is entitled "On the Dropsy of the Ducts of Glands, *a disease hitherto unknown;*" but it may be fairly questioned how far this name is applicable to the appearances which he has described. The ducts of various glands are liable to obstruction from various causes; the gall-ducts and the ureters are often found obstructed by concretions or by adhesive inflammation, and the consequences are enlargement and dilatation of the ducts. When such obstruction takes place in the gall-ducts, jaundice is an uniform symptom; the ducts swell and dilate, and one of two consequences ensue,—either the bile continues to be secreted till the ducts are stretched to the uttermost, and, being then no longer able to resist the pressure, give way, and effuse their contents into the abdominal cavity; or the ducts are strong enough to resist the pressure, and continue to dilate sometimes to an enormous extent, and the pressure, reacting upon the hepatic ducts, dilate them also throughout the whole substance of the liver.

When this state continues for any length of time the secretion of the bile is altered, and it sometimes degenerates into a watery serous fluid; or, according to Dr. Albers, there ensues dropsy of the gall-ducts. This serous bile has never been observed by him except in cases of obstruction of the ducts; and hence he ascribes it to a diseased condition of their mucous surface. It seems to us, however, to be altogether dependent upon the state of the parenchyma of the liver, which state may certainly follow as a consequence of obstruction of the ducts; for we have two cases recorded by Andral, in his Clinique Medicale,* in which the gall-ducts contained serous bile, without any trace of stricture or obstruction being observable in the ducts. Neither is dilation of the gall-ducts† nor serous bile discoveries of Albers; but our author glories in having been the first to assign them their true place in systems of nosology.

When the ureters are obstructed, either by calculi or inflammation, and the urine continues to be secreted, a gradual dilatation, first of the ureters themselves, and then of the pelves and calyces, takes place, so as in some instances to convert the kidney into one immense sac. It is probable that the chemical nature of the urine is at the same time altered; but of this, as in general only one kidney suffers, it is difficult to adduce positive proof. This state Dr. Albers calls *Dropsy of the ureters;* and in the same way we have dropsy of the parotids, of the testis, pancreas, &c., the dropsy (if such it may be termed,) being evidently an effect or symptom of the original disease, and not the disease itself.

The second observation contains a case of dilated thoracic duct, and its object is to support the view that the thoracic duct communicates with the venous system by frequent anastomosis, and not solely by the opening into the left subclavian vein. We noticed the subject in our Fourth Number, p. 535.

The subject of the next article is *Thymic Asthma;* but we have elsewhere spoken so fully on this affection, that we have only to remark that it is treated by Albers in an able and comprehensive manner. He does not view it as a peculiar disease, but considers its symptoms as common to a variety of affections of the circulatory and respiratory apparatus.

The fourth paper is of considerable merit, but affords another instance of the rashness with which Dr. Albers draws general conclusions from limited experience.

"There is," he says, "a form of softening of the spinal marrow, which commences in the grey matter, to all appearance in the neighbourhood of the central fissure, and extends slowly outwards, affecting the white substance, but much more rapidly upwards, confined to the centre of the cord. It generally commences in the cauda equina, and advances slowly upwards; after a lapse of several years, reaches the medulla oblongata, or extends even into the ventricles of the brain. This partial softening is sufficiently characterized by its peculiar progression from below upwards, but its symptoms and the very gradual and tedious course of the disease are also remarkable." (P. 73.)

In illustration of this form of disease, he gives the history of two cases, in the first of which the following morbid appearances were found on inspection of the vertebral canal.

"The dura mater appeared healthy, but between it and the arachnoid were contained two drachms of a reddish fluid. The arachnoid was partially thickened in the lumbar and lower half of the dorsal region, opaline, and contained three ossified scales of the size of peas. The pia mater was thick and firm, almost tendinous and stri-

* Vol. iv. p. 155. Cases xi. and xii.

† Vide Cruveilhier, Dictionnaire de Med. et Chirurg. prat. Art. Foie, maladies du.

ated, but red and vascular. On cutting into the cauda equina, the softened medulla flowed through the cut as a mere pulpy mass of a yellowish grey colour. Towards the fourth dorsal vertebra, a thin layer of white matter, enclosing the softened greyish pulp, adhered to the pia mater, and gradually increased in thickness till the medulla acquired its normal condition in the region of the third cervical vertebra." (P. 80.)

In the second case, the dura mater was healthy; the arachnoid adhered firmly to the pia mater, and contained ossified scales and white opaline patches. The pia mater was very firm, and adhered strongly to the medulla. In the lumbar region, the whole thickness of the spinal cord was reduced to pulp, but higher up the softening gradually retracted towards the centre, till it ceased about the fourth cervical vertebra.

The following symptoms, deduced from these two cases, are given by Dr. Albers as characteristic of central softening of the spinal marrow:— The course of the disease is very slow and tedious, and hence the accompanying paralysis is of very gradual accession, and of very long duration. Sensibility is affected at an earlier period of the disease than motion; and the patient complains of severe pains in the paralyzed limbs, a feature which always accompanies this variety of softening. The lower extremities assume a peculiar shape, analogous to that of the clubfoot, and the sensation as if a tight band were drawn round the abdomen, is experienced very early in the disease. Lastly, gangrene is said to be a rare occurrence in cases of this kind.

It is stated by Dr. A. that central softening of the spinal cord has hitherto been overlooked. This is only partially the case: Andral, Ollivier, and many other authors have noticed it; but we are not aware of any peculiar or characteristic symptoms having been ascribed to it. It could not possibly have been overlooked by Ollivier, who supposes that, in every case of softening of the spinal cord, the change begins in the centre; and, as Dr. A. himself quotes a case from Ollivier, we suspect that it is the importance of the variety which he thinks has been overlooked. Admitting that, in the majority of cases, softening commences in the grey matter, we are not disposed to agree with Ollivier in considering this as always the case; and there are no sufficient grounds, we submit, for denying the occurrence of primary softening of the white matter of the cord. Original softening of the white substance of the brain is generally allowed, and, reasoning from analogy, we should expect a like affection of the white matter of the medulla.

Whether the softening in the cases recorded by Dr. Albers commenced originally in the grey or white substance, we believe it impossible, in the present state of our knowledge, to determine with certainty; but the point is at once assumed as settled by our author, without even the shadow of proof being deemed necessary. The symptoms noted by him as characteristic of central softening are of little value in determining the presence of this specific variety: they might apply equally well to any case of this affection, and to many other diseases of the spinal cord. Dr. A. describes central softening as a *species* of medullary softening: he does not, therefore, agree with Ollivier in the opinion that it always commences in the centre; and he has said nothing of the symptoms which he regards as characteristic of the other varieties of softening, which we might contrast with those advanced as peculiar to central softening. We cannot afford space to enquire in how far the greater softening of the grey matter in Dr. Albers's cases might be allowed to support his views, or in how far they might be subverted by taking into consideration the morbid condition of the membrane of the cord: there is evidently too much speculation based upon a very narrow foundation.

This essay is followed by several well-compiled papers on various subjects; and the volume concludes with an important and interesting article, giving an account of the Structure and Functions of the Glands of the Intestinal Canal. Since the origin of the Broussaisian school, the intestinal glands have performed an important part in pathological history, and they are well known under the title of Brunner's and Peyer's glands. Attempts have been made from time to time,* but without attracting much attention, to enquire minutely into their intimate structure: the standard works of Andral, Louis, and Abercrombie are silent upon this point; and, although Billard has been more minute than any of these authors in his description of them, he has added but little to our knowledge. The subject, however, was some time since taken up by Dr. Boehm, of Berlin, whose excellent work on the subject† we analyzed at great length in our Second Number, and illustrated our analysis by copies of the original plates, (see Vol. I. p. 521.) As Dr. Albers coincides almost entirely with Dr. Boehm respecting the anatomy of these parts, we shall content ourselves with referring our readers to our previous article on the subject. Dr. Albers's essay adds little to what was already supplied by Boehm, but it is of importance as corroborative of his views.

Peyer's patches vary exceedingly both in number and degree of development; they are sometimes so indistinct as to be scarcely perceptible, and at other times they form prominent projecting surfaces. They are greatly under the influence of disease, not only in point of development, but also in point of number. Scoutteten‡ holds not only that their number is variable in different individuals, but that they may be increased or diminished by individual habit of body. Fohmann and Von Halen§ are of the same opinion, and maintain that various conditions of the body may produce a development of Peyer's patches, and cite pregnancy as a case in point. It is a generally received opinion that they are more frequent in youth than in old age, and the idea that their number and development are influenced by disease is by no means uncommon.

We are not prepared to say in how far the views of Boehm and Albers respecting the intestinal glands may be considered as correct. The subject is one, however, well deserving the attention of the physiologist and pathologist; and it appears to us a matter of surprise, considering the great importance which has for some years been attached to diseases of the intestinal mucous surface, that such an investigation had not been sooner entered upon; and, we feel assured that a work either in confirmation or in refutation of the views of the authors above mentioned would render an essential service to the science of medicine.

* Anatomisch-physiologische Abhandlungen, von Karl A. Rudolphi. Berlin, 1802. —Lieberkühn, Dissertationes Anatomicæ, curâ J. Sheldon. London, 1782.

† De Glandularum Intestinalium structura penitiori commentatio anatomica. Dissert. Inaug. Anatom. Auctore Lud. Boehm.—Berolini, 1835.

‡ Journ. Complement. du Diction. des Sciences Med., 1829.

§ De Glandulis Conglomeratis Specimen Inaugurale. Leod. 1830.

